# Anti-Tumoral Treatment with Thioredoxin Reductase 1 Inhibitor Auranofin Fosters Regulatory T Cell and B16F10 Expansion in Mice

**DOI:** 10.3390/antiox14111351

**Published:** 2025-11-11

**Authors:** Michael Y. Bonner, Tamas Vancsik, Ana Oliveira-Coelho, Pierre Sabatier, Christian M. Beusch, Kejsi Zeqiraj, Carolin Svensson, Roman A. Zubarev, Elias S. J. Arnér, Rikard Holmdahl

**Affiliations:** 1 Division of Medical Inflammation Research, Department of Medical Biochemistry and Biophysics, Karolinska Institutet, 171 65 Solna, Sweden; michael.y.bonner@gmail.com (M.Y.B.); ana.coelho@ki.se (A.O.-C.); k.zeqiraj@gmail.com (K.Z.); carolin.svensson@ki.se (C.S.); 2 Division of Biochemistry, Department of Medical Biochemistry and Biophysics, Karolinska Institutet, 171 65 Solna, Sweden; tamas.vancsik@usz.ch (T.V.); elias.arner@ki.se (E.S.J.A.); 3 Division of Chemistry I, Department of Medical Biochemistry and Biophysics, Karolinska Institutet, 171 65 Solna, Swedenchristian.beusch@gmail.com (C.M.B.); roman.zubarev@ki.se (R.A.Z.); 4 Department of Selenoprotein Research and the National Tumor Biology Laboratory, National Institute of Oncology, 1122 Budapest, Hungary

**Keywords:** auranofin, regulatory T cells, thioredoxin reductase 1, reactive oxygen species, TRi-1, NRF2, B16F10, NOX2, NCF1, antigen presenting cells

## Abstract

Auranofin, an FDA-approved antirheumatic drug and thioredoxin reductase 1 (TXNRD1) inhibitor, has demonstrated anti-tumoral properties, but its immunological effects are not well characterized. Here, we report that auranofin unexpectedly promotes regulatory T cell (Treg) expansion. In a B16F10 melanoma model, auranofin treatment increased lung tumor coverage, IL-10 serum levels, and FOXP3^+^CD44^+^CD4^+^ T cell frequencies. It also altered the proportion of antigen-presenting cells (APCs), increasing B cells and reducing dendritic cells. To test whether Treg expansion occurs independently of tumor antigens, we stimulated T cells ex vivo in lymph node cultures from naïve mice using anti-CD3/CD28, with or without auranofin. Auranofin increased Treg frequency in these cultures, as well as in treated human PBMCs. Similar effects were observed with the TXNRD1 inhibitor TRi-1, suggesting a ROS-dependent mechanism. Using mice with conditional expression of neutrophil cytosolic factor 1 (NCF1), we found that both TXNRD1 inhibition and APC-specific NCF1-NOX2-ROS expression enhanced tumor burden and Treg expansion. Alternatively, sorted T cells from mice harboring conditional TXNRD1 knockouts showed reduced FOXP3 and GITR expression in the naïve state and reduced tumor burden when challenged with B16F10. These data suggest TXNRD1 inhibitors likely drive Treg expansion by elevating ROS levels in APCs during T cell priming and less by intrinsic Treg TXNRD1 blockade. Our findings reveal a paradoxical immunosuppressive effect of TXNRD1 inhibitors that may contribute to their limited efficacy in immunocompetent cancer models. This work provides mechanistic insight and underscores the need to consider Treg-mediated immune suppression when designing TXNRD1-targeted therapies.

## 1. Introduction

Cancer remains a major health challenge, with many patients not benefiting from current treatments. Thus, new therapeutic strategies are urgently needed. One promising approach is repurposing approved drugs like auranofin [1]. Auranofin (Ridaura^®^), an FDA-approved disease-modifying antirheumatic drug (DMARD), is prescribed to patients unresponsive to first-line therapies such as methotrexate and anti-TNFα [2]. Although approved in 1985, its precise immunomodulatory mechanism remains unclear [2]. Initial studies indicate that auranofin impairs T cell activation and proliferation by depleting glutathione [3,4]. Its gold moiety binds non-specifically to conserved active sites of redox enzymes like thioredoxin reductase 1 (TXNRD1) [5,6] and glutathione peroxidase 1 [7], which regulate reactive oxygen species (ROS), including hydrogen peroxide [8]. Inhibiting TXNRD1 disrupts redox balance, leading to ROS accumulation and compensatory NRF2 activation [9]. Moreover, specific TXNRD1 inhibitors appear to further elevate intracellular ROS levels, implicating this pathway in auranofin’s immunomodulatory effects [10].

Cancer cells, being highly metabolically active, produce significant ROS levels, which can limit their growth. Tumors with elevated ROS levels depend on increased NRF2 activation, making them sensitive to further increases in ROS levels through TXNRD1 blockade [8,11]. Auranofin-mediated TXNRD1 inhibition has shown anti-tumor efficacy in multiple cancer models both in vitro and in vivo [12,13,14]. Short interfering RNAs (siRNAs) studies targeting TXNRD1 further highlight its critical role in tumor progression [15]. Over the past decade, clinical trials have explored auranofin’s potential in treating non-small cell lung cancer, lung carcinomas, ovarian cancer, glioblastomas, and leukemias. However, the clinical impact of these trials (e.g., NCT01747798, NCT01737502, NCT02770378, NCT01419691) remains unclear. Notably, recent trials (NCT03456700, NCT1737502) combining auranofin with sirolimus in PKCι-positive tumors also reported no clear therapeutic benefit.

This highlights a gap in our understanding of auranofin’s anti-cancer activity and our need to better understand how TXNRD1 inhibition affects not only tumor-intrinsic redox states but also the immune microenvironment. Existing research has shown auranofin’s benefits in leukemia models and immunocompromised mouse strains, but comprehensive studies in immunocompetent models, which have nuanced differences, are limited. Both auranofin and the more specific TXNRD1 inhibitor, TRi-1, suppress tumor development in PyMT-MMTV mice that spontaneously develop malignant breast tumors [12], as well as in other triple negative breast cancer models, particularly if combined with anti-PD-L1 antibody [12,16]. While these studies use immunocompetent mouse strains such as the FVB [17] and BALB/c, respectively, both have notable immunological limitations [18]. The FVB strain carries mutations in the T cell receptor β Variable 8 chain (*Tcrb-V8*) [19] and shows reduced tumor immunoediting compared to PyMT-MMTV mice backcrossed to the C57BL6 strain [20]. BALB/c mice display significantly reduced anti-tumoral toxicity from natural killer cells (NK) [21] and enhanced Treg suppressive activity compared to C57BL6 mice [22], likely contributing to increased susceptibility to grafts, including tumors, from MHC-mismatched strains [21,23]. Although a few other studies exist in immunocompetent mice [13,24,25], on balance, auranofin’s anti-cancer activity demonstrated in studies on immunocompromised and blood cancer models [1], as well as in vitro studies, has yet to fully translate to immunocompetent models.

Auranofin’s REDOX modulatory activity on anti-tumor immunity remains underexplored but is significant based on key findings. First, elevated ROS levels in immune cells, such as increased NOX2-ROS production during activation, may suppress anti-tumor responses. Conversely, mice deficient in NOX2 due to NCF1 mutations show reduced susceptibility to immune-dependent tumors like the B16F10 melanoma [26]. Second, NRF2 activation, a hallmark of TXNRD1 blockade, suppresses inflammatory responses in immune cells [27,28,29,30,31]. Notably, NRF2 activation via KEAP1 mutations suggests it can be immunosuppressive even in the absence of upstream ROS signaling [32].

This study investigates the immunological impact of TXNRD1 blockade through auranofin treatment of the B16F10 model in immunocompetent mice, leading us to focus on its effect on Treg populations in both murine models and human cells. Our findings suggest that TXNRD1 blockade may enhance tumor progression in vivo through increased immune suppression, despite consistent anti-cancer activity in vitro and in immunocompromised models. By elucidating these mechanisms, our research seeks to advance our understanding of redox modulators such as auranofin.

## 2. Materials and Methods

### 2.1. Study Design

We initially operated under the hypothesis that TXNRD1 inhibitor treatment will reduce tumor burden in the B16F10 lung colonization model. After initial results did not support this hypothesis, we began to operate under a working hypothesis that TXND1 inhibitors promote tumor progression through immune suppression. We evaluated this hypothesis through lines of inquiry into Treg expansion by TXNRD1 inhibitors or H_2_O_2_ and through an evaluation of APC NOX2/NCF1-derived ROS contributions. Experimental conditions are described below, with all reagents and resources, also conveniently listed in Table A1, Table A2 and Table A3.

### 2.2. Cell Culture

All cells, including B16F10 obtained directly from the American Type Culture Collection (ATCC CRL-6475), were cultured in a humidified 37 °C, 5% CO_2_ incubator. B16F10 were maintained at a passage less than 15 in 10% FBS (heat inactivated) (ThermoFisher Scientific, 11573397, Waltham, MA, USA) in DMEM + Gluta MAX (Gibco , Thermo Fisher Scientific, cat. 31966-021, Waltham, MA, USA)) supplemented with penicillin and streptomycin. Cells were expanded and cryopreserved in 10% DMSO from passage 4 to 9 and thawed between 1 and 2 weeks prior to studies. Cells tested negative for mycoplasma using the MycoAlert^®^ MycoPlasma Detection Kit (Lonza, LT07-118, Basl, Switzerland).

Primary cultures of mouse splenic leukocytes or human PBMCs were cultured in 10% FBS (heat inactivated) (Thermo Scientific 11573397) RPMI 1640 GlutaMAX (Gibco, cat. 61870044), 50 µM 2-Mercaptoethanol (50 mM stock) (Thermo Fisher Scientific, cat. 31350010) supplemented with penicillin and streptomycin.

### 2.3. SRB NCI-60 Screen for IC50

B16F10 cells were cultured and treated in a modified version of the NCI-60 protocol [33]. A 100 µM stock solution of each Txnrd1 inhibitor was prepared in DMSO and diluted to the indicated concentration with growth media, with the control containing the highest concentration of DMSO corresponding with the highest inhibitor concentration. Briefly, 1 × 10^4^ cells were plated in 100 µL of growth media and incubated for 24. Cells were then washed and provided with 100 µL of growth media with treatment at indicated concentrations or control and incubated for 24 or 48 hrs. Cells were subsequently washed, fixed with cold TCA (final concentration 10% in H_2_O), and incubated at 4 °C for 1 h. The cells were washed 5× with tap water and allowed to air dry. The cells were then stained with 100 µL of Sulforhodamine B (SRB) solution (4% *w*/*v*) in 1% acetic acid to each well and incubated for 10 min at room temp. The cells were washed 5× with cold 1% acetic acid and allowed to air dry. Finally, 100 µL of 10 mM trizma base was added to solubilize bound dye before reading the absorbance in a plate reader at 515 nm.

### 2.4. qPCR of Genes Downstream of NRF2 Activation

Eurofin-PCR primers were designed and BLASTed at NCBI Primer-BLAST (https://www.ncbi.nlm.nih.gov/tools/primer-blast/index.cgi?LINK_LOC=BlastHome (accessed on 5 April 2023)). Primers were subjected to a melting curve analysis, gel analysis, and optimized for each indicated gene. RNA from three independent B16F10 early passage cultures (p15 or less) was isolated using the Qiagen (Venlo, The Netherlands) Qiashredder columns and RNA easy kit. RNA quality and abundance were quantified on a Thermofisher Nanodrop. cDNA was produced using the BioRad (Hercules, CA, USA) iScript Kit following the manufacturer’s recommendations. Rt-PCR was performed using a SYBR Green probe and acquired on a Cfx BioRad Real time instrument using the optimized Eurofin-PCR primers (Luxembourg).

### 2.5. Animals

Founders of C57/B6N.Q/rhd, denoted as **B6NQ**, or C57/B6N.Q. NCF1/rhd, denoted as Ncf1*^/^*, mice carrying a point mutation in the *Ncf1* gene (*m1j*) resulting in a loss of function in the NOX2 complex [34], were backcrossed and maintained by the Holmdahl laboratory as inbred lines (rhd). The B cell specific ROS strain B6N.Q.Mb1-cre^+^.TN3, denoted as **TN3.Mb1-Cre**, or dendritic cell (DC) specific ROS strain B6N.Q.CD11c-Cre^+^.TN3, denoted as **TN3.CD11c-Cre** [35], were created by, respectively, backcrossing the transgenic strain Mb1-Cre^Tg^ (stock 020505) or CD11c-Cre^Tg^ (stock 018967) from Jackson Laboratory with our Ncf1*^/^* strain and then crossing them into our targeted *Ncf1* knock-in strain B6N.Q.*Ncf1^TN3^*^/^*^TN3^*, denoted as **TN3** [36]. The TN3 mice express both the low ROS-producing NCF1^153M^ allele and the high ROS-producing NCF1^T153^ allele, with the latter in an inverted orientation, mirroring the mutation shown to regulate oxidative burst and arthritis severity in rats. Crossing the B6NQ.Mb1-Cre.NCF1 and the TN3 mice strains resulted in the deletion of the low ROS NCF1^153M^ allele and the inversion of the high ROS NCF1^T153^ allele to the proper orientation, which led to the expression of the NCF1 protein in *Mb1*-expressing cells, mainly B cells at different stages of differentiation. Analogous results occur by crossing TN3 and B6NQ.CD11c-Cre.NCF1, allowing expression of the NCF1 protein in *CD11c*-expressing cells, mainly DCs and pDCs. For studies evaluating thioredoxin reductase conditional knockouts, Txnrd1^fl/fl^ mice (stock 028283), reposited at Jackson Lab, were bred with CD4-Cre^Tg^, originating from (stock 022071) from Jackson Laboratory, and backcrossed onto C57/B6N/rhd, denoted as **B6N**, for more than 15 generations, resulting in B6N.Txnrd1^fl/fl^ CD4-Cre^+^, denoted as **Txnrd1.CD4-Cre**.

Sex- and age-matched littermate controls of approximately 10–16 weeks old (either male or female) mice were randomized and distributed 4–5 mice/cage in blinded experiments. They were housed under specific pathogen-free (SPF) conditions in individually ventilated cages. Cages were supplemented with wood shaving bedding in a climate-controlled environment with a 12 h day/night cycle. These studies were approved by the Stockholm regional animal ethics committee of Sweden in protocols (N288/15 or in the renewed version 16974-2020).

### 2.6. In Vivo Tumor Studies

Mice were intravenously injected with early passage 1.5 × 10^5^ B16F10 cells (ATCC CRL-6475), maintained at a passage less than 12, and suspended in 100 μL of phosphate-buffered saline (PBS, 1×; Life Technologies Europe BV, Stockholm, Sweden). Mice were treated as previously described [12], with 100 µL of vehicle composed of 10% inhibitor stock (10 mg inhibitor/100 µL of DMA (N,N-Dimethylacetamide, Sigma, Burlington MA, USA) dissolved into a 1:1 PEG 400 (Merck, 8.17003.1000, Darmstadt, Germany): Cremophor (Sigma-Aldrich, C5135-500G, Burlington, MA, USA) mixture) in 90% PBS. Treatment groups received 1.2 mg/kg of auranofin or 10 mg/kg of TRi-1, prepared by pre-dissolving in the 10% 1:1 PEG 400: Cremophor, containing inhibitor, prior to addition of 90% PBS. Injection cocktails were prepared fresh the day of injection and administered IP. After no more than 28 days, the mice were anesthetized, bled, and sacrificed. Their lungs perfused with PBS, tumor biopsies were collected and snap frozen, spleens harvested and lungs photographed from two different sides using a Canon (Ota-ku, Tokyo, Japan) Quick-shot. Tumor burden was typically assessed and quantified using QuPath [37] analysis software version 0.3.2 from the two photographs of each sample, or as described in the results.

### 2.7. For Adoptive Transfer of DCs

Bone marrow derived DCs were derived from isolated bone marrow of B6NQ mice and cultured for 14 days in FLT3L 100 ng/mL (Biolegend 550704, SanDiego, CA, USA) + DLL1 (R&D systems 5026-DL-050, Minneapolis, MN, USA). BMDCs were washed and resuspended in PBS at a concentration of 2 × 10^7^ per ml. B16F10 tumor-bearing mice (day 1 post-inoculation) and NCF1 mice were injected I.V. with 100 µL or 2 × 10^6^ cells.

### 2.8. Serology via MSD

Serum samples from tumor bearing mice treated with auranofin, TRi-1, or vehicle control were compared to serum samples from naive mice treated with the vehicle control. The sample were incubated using the Meso Scale Diagnostics (MSD) (Rahway, NJ, USA) U-PLEX Custom Biomarker Group 1 (cat. K15069M-2) according to the manufacturer’s instructions. The data was acquired using a MESO QuickPlex SQ 120MM.

### 2.9. Proteomics Sample Preparation

Snap frozen lung tissue foci biopsies from B16F10 mice treated with auranofin, TRi-1, or control were lysed in 1% SDS, 8 M Urea, and 50 mM Tris (pH 8.5). The tissue was homogenized using a Branson (Danbury, CT, USA) probe sonicator for 45 s (3 s on, 3 s off, 30% amplitude). Protein concentration was determined using the Pierce bicinchoninic acid protein assay kit (Thermo Fisher Scientific) following the manufacturer’s protocol. Equal amounts of protein from each sample were reduced with 5 mM DTT for 1 h, followed by alkylation in the dark with 15 mM IAA. Methanol/chloroform precipitation was performed as follows: three volumes of methanol were added to the samples, followed by one volume of chloroform and three volumes of water. The samples were vortexed between each addition and then centrifuged at 20,000× *g* for 10 min at 4 °C. The aqueous phase was removed, and the protein pellet was washed with one volume of methanol, vortexed, and centrifuged at the same speed. After discarding the liquid, the protein pellet was air-dried. The air-dried pellets were resuspended in 8 M Urea and 20 mM EPPS (pH 8.5), and diluted with 20 mM EPPS (pH 8.5) to a final concentration of 4 M Urea. Lysyl endopeptidase (LysC) digestion was performed overnight at room temperature at a 1:100 (*w*/*w*) LysC to protein ratio. The following day, samples were diluted fourfold with 20 mM EPPS (pH 8.5) to reduce the Urea concentration to 1 M. Tryptic digestion was then carried out for 6 h at room temperature (RT) with a 1:100 (*w*/*w*) trypsin to protein ratio. TMTpro16 labeling was performed for 2 h at RT by adding 0.2 mg of reagent dissolved in dry ACN, resulting in a final ACN concentration of 20%. The reaction was quenched by adding triethylamine to a final concentration of 0.5%, followed by a 15 min incubation at RT. The labeled samples were combined, resulting in one pooled sample per replicate, with each temperature condition included. The pooled samples were acidified to pH < 3 using TFA, desalted with Sep-Pak (Waters, Milfor, MA, USA), and vacuum-dried overnight using a miVac DNA concentrator (Genevac, Ipswich, UK). Peptides were fractionated using a high-pH reversed-phase chromatography method on a Dionex Ultimate™ 3000 RSLCnano System (Sunnyvale, CA, USA). The peptides were separated on a C18 XBridge Peptide BEH 25 cm column (2.1 mm internal diameter, 3.5 µm particle size, 300 Å pore size; Waters) using a gradient with buffer A (20 mM NH_4_OH in water) and buffer B (100% ACN). The gradient started with 1% buffer B, increasing to 23.5% over 42 min, then to 54% B for 9 min, followed by 63% B for 2 min, holding at 63% B for 5 min, and finally returning to 1% B for 7 min. The resulting 96 fractions were concatenated into 12 fractions and vacuum-dried using a miVac DNA concentrator.

### 2.10. Mass Spectrometry Analysis

The samples were resuspended in 2% acetonitrile (ACN) and 0.1% formic acid (FA) (buffer A) and injected into an UltiMate 3000 UPLC autosampler or EASY-LC (Thermo Scientific) coupled to an Orbitrap Fusion Lumos Tribrid mass spectrometer (Thermo Scientific). The peptides were loaded on a trap column (Acclaim PepMap 100 C18, 100 μm × 2 cm) and separated on a 50 cm long C18 Easy spray column (Thermo Scientific).

Chromatographic separation was achieved using the following gradient: 4–24% of solvent B (98% ACN and 0.1% FA) in 120 min, 24–34% in 30 min, 34–95% in 3 min, and 5 min of 95%, before equilibration for 7 min at 4% with a flow rate of 300 nl×min^−1^. For data collection, the mass spectrometer operated in positive polarity using a data-dependent acquisition mode. The cycle time was 3 s and consisted of one full scan with a resolution of 120,000, covering the range from 375 to 1400 Th. Automatic gain control was set to 1 × 10^6^ with a maximum injection time of 50 ms. Triggered MS/MS scans were recorded with a resolution of 50,000, AGC of 25,000, maximum injection time of 54 ms, isolation window of 0.7 Th, and normalized collision energy (NCE) of 35%. Only peptides with a charge from 2+ to 5+ were selected, and dynamic exclusion was set to 60 s.

### 2.11. Proteomics Analysis and Statistical Analysis

Raw data files were searched on a custom-modified version of MaxQuant (2.0.1.0) recognizing TMTpro as an isobaric label. For peptide searches, acetylation of the N-terminal and oxidation of methionine were selected as variable modifications, whereas carbamidomethylation of the cysteine was selected as a fixed modification. Trypsin with up to two missed cleavages was set as protease, and the spectrum was searched against the SwissProt *Mus musculus* database. The “match between run” feature was enabled. The FDR was set to 0.01 for both peptide and protein identification. For all other parameters, default settings were used.

Data analysis and plots were produced using R software version 4.3.3. Only proteins with no missing values and at least two quantified peptides were considered. Individual protein abundances were normalized by the sum of all protein abundances in the corresponding sample. Auranofin or TRi-1 treated samples were compared to the control treated samples using a two-tailed unpaired *t*-test. Data are presented as mean ± standard error of the mean, unless otherwise stated. *p*-values lower than 0.05 were considered statistically significant. Separate GO pathways enrichment pathways analysis using DAVID version 6.8 Function Clustering Annotation of all proteins differentially for (1) downregulated auranofin vs. control, (2) upregulated auranofin vs. control, (3) downregulated TRi-1 vs. control, (4) and upregulated TRi-1 vs. control. Statistical significance was determined by Bonferroni correction, with *p* value < 0.05 deemed as significant.

### 2.12. Ex Vivo Murine Treg Expansion Studies

Lymph nodes from sex- and age-matched littermate controls of approximately 10–16 weeks old (either male or female) mice were harvested and mashed through a 40 µM mesh filter (Corning 431750, Corning, NY, USA) and seeded at approximately 1–2 × 10^6^ cells per well in a 96-well round bottom plate (Falcon 353077). The cells were cultured in RPMI 1640 media (Gibco 11875093) containing 10% heat-inactivated FBS and 50 µM 2-mercapto-ethanol (Gibco 21985023). Cells were stimulated for 72–96 h with 1 µg/mL anti-CD3ε (BD Biosciences, clone 145-2c11) and anti-CD28 (BD Biosciences, clone 37.51, Franklin, NJ, USA). Treg polarization was further stimulated with 5 ng/mL or 645 IU/mL Recombinant Murine IL-2 (PeproTech 212-12, Cranbury, NJ, USA) and TGFβ or with TXNRD1 inhibitors suspended in DMSO, namely auranofin or TRi-1 at indicated concentrations, usually 0.1 μM.

### 2.13. Ex Vivo PBMC Treg Expansion Studies

Healthy donor buffy coat (*n* = 4, female) was ordered from Karolinska University Hospital in accordance with ethical permit Dnr 2020-05001. PBMC isolation was performed by washing with PBS and using a ficoll gradient Ficoll-Paque from Cytiva, Uppsala, Sweden. Total PBMCs were seeded at approximately 1–2 × 10^6^ cells per well in a 96-well round bottom plate (Falcon 353077). The cells were cultured in RPMI 1640 media (Gibco 11875093) containing 10% heat-inactivated FBS and 50 µM 2-mercapto-ethanol (Gibco 21985023). Cells were stimulated for 72–96 h with 1 µg/mL anti-CD3ε (BD Biosciences 550367, clone HIT3a) and anti-CD28 (BioLegend 302902, clone CD28.2). Treg polarization was further stimulated with 5 ng/mL or 118 IU/mL of recombinant human IL-2 (BioLegend 589102) and recombinant human TGFβ (BioLegend 781802) or with TXNRD1 inhibitors suspended in DMSO, namely auranofin or TRi-1 at indicated concentrations, usually 0.1 μM.

### 2.14. Intracellular Staining for Cytokines

Human PBMCs were stimulated with 100 ng/mL of PMA and 1 μg/mL of ionomycin in the presence of 5 μg/mL of brefeldin A (BFA) for 4 h at a humidified 37 °C, 5% CO_2_ incubator. The stock solutions of PMA, ionomycin (ThermoFisher, Catalog Number I24222), and BFA (ThermoFisher, catalog no. B7450) were prepared with dimethylsulfoxide (DMSO, Sigma-Aldrich Co., CAS no. 67-68-5). For intracellular cytokine staining, cells were fixed and permeabilized using BD cytofix/cytoperm solution (BD Biosciences, catalog no. 554714). Samples were acquired using an Attune flow cytometer (Thermo Fisher Scientific, Waltham, MA, USA).

### 2.15. Flow Cytometry Analysis

Single-cell suspensions derived from cultured B16F10, murine lymph nodes and spleens from naïve and tumor-bearing mice, or human PBMCs were acquired and analyzed by flow cytometry using an Attune NxT Acoustic Focusing Cyometer (Lasers: BRVX) (Thermo Fisher). The workstation is managed by an OPTIXE2 Microsoft Windows 7 Professional (Thermo Fisher Scienific, Waltham, MA, USA), and the data were analyzed using the FlowJo software version 10.10 (Becton Dickinson & Company (BD)). Cell sorting was achieved using a BD ARIA ii, with a 70 µm nozzle. 

The cell samples were stained with a LIVE/DEAD^®^ fixable AQUA or APC-Cy7 dead cell stain (Thermo Fisher, catalog no. L10119) according to the manufacturer’s instructions and blocked with an in-house anti-mouse CD16/CD32 Fc block. Extracellular antigens were stained, covered, for 25 min at 4 °C in RPMI with 10% fetal bovine serum (FBS, Gibco, Thermo Fisher, catalog no. 26140079). 

### 2.16. Measuring Intracellular ROS via DHR Staining

B16F10 cells were treated for 12 h with auranofin, TRi-1, or control at the indicated concentrations in their growth medium and incubated at 37 °C, 5% CO_2_. Cells were washed and stained for 20 min at 37 °C, 5% CO_2_ in pre-warmed growth medium containing a final concentration of 3 μM DHR (ThermoFisher, Catalog Number D23806) after staining for cell surface markers and live/dead staining. Finally, the cells were washed and acquired by flow cytometry in 200 µL of PBS.

For immune cells, cells were also incubated with 3 μM DHR for 10 min after staining for cell surface markers and live/dead staining, but also underwent stimulation with 200 ng/mL PMA (Millipore Sigma-Aldrich, P8139) or anti-IgM, (µ chain specific) (for B cell receptor only) (Jackson ImmunoResearch, West Grove, PA, USA) for 20 min at 37 °C before data acquisition by flow cytometry.

### 2.17. Statistics and Reproducibility

Statistical analyses were performed with Graph Prism software, version 10 (GraphPad Software, San Diego, CA, USA). Generally, but not always, non-parametric tests were used to minimize the influence of extreme values. Where outliers were suspected, both the ROUT (Q = 1%) and Grubbs’ test (alpha = 0.05) were employed. No outliers were identified in any data set. Results from non-parametric tests such as the Mann–Whitney U test and Kruskal–Wallis test are shown as median ± interquartile range. Parametric tests were used where the need for statistical power outweighed concern for an extreme value’s influence. Given no data set was determined to have an outlier and that many studies had a small sampling size (<12), majority of the tests performed were parametric. Results from all parametric tests are shown as geometric mean ± geometric SD, as most data sets contain some lognormal distributions. In all cases where multiple hypothesis tests occurred, a correction was also employed. All tests were performed with either a 95% confidence interval or a False Discovery Rate (FDR) of Q = 0.05. Specific tests are specified in the respective figure legends. *p* value < 0.05 was considered as significant: * *p* < 0.05, ** *p* < 0.01, *** *p* < 0.001, and **** *p* < 0.0001. Likewise for FDR, *q* < 0.05 was considered a discovery and labeled significant.

## 3. Results

### 3.1. TXNRD1 Inhibitors Elevated ROS Levels, Activate NRF2, and Kill B16F10 Cells In Vitro

**Figure 1:**
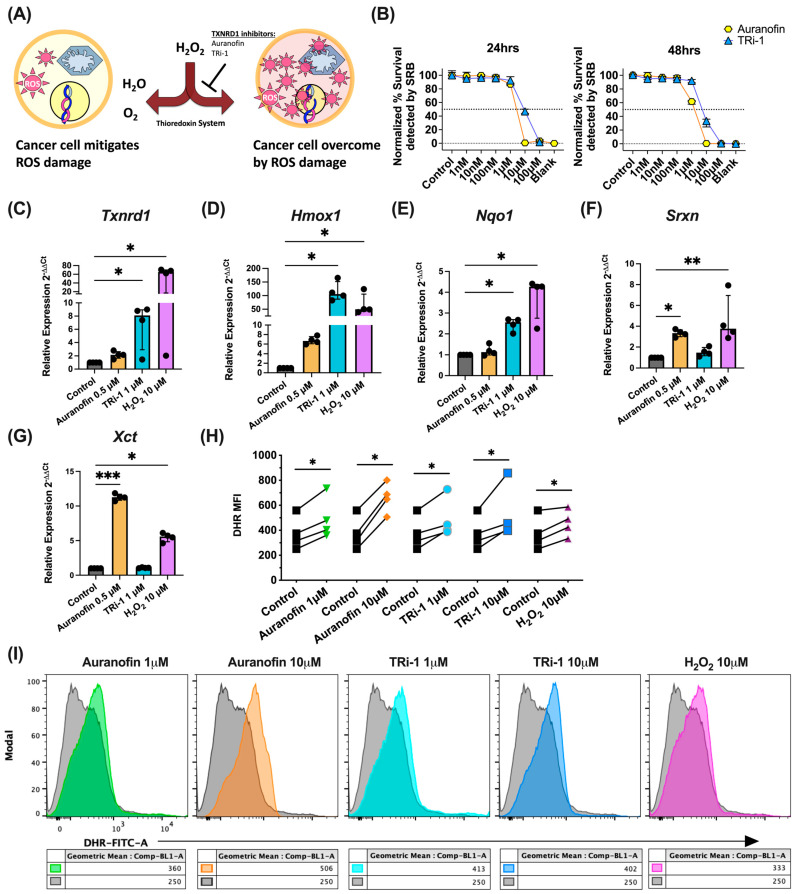
Anti-cancer TXNRD1 inhibitors increase Nrf2 activation via elevated intracellular ROS levels. (**A**) The prevailing hypothesis in the field postulates that TXNRD1 inhibitors such as auranofin may effectively target cancer cells by inhibiting the thioredoxin system, critical to innate antioxidant cellular response, allowing a toxic accumulation of reactive oxygen species such as hydrogen peroxide. (**B**) A 24 h and 48 h titration determining the in vitro IC_50_ (dashed line) of auranofin and TRi-1 on the B16F10 melanoma cell line via the detection of SRB-protein staining at 515 nm, (median, 95% CI). SD reflects the technical variance from *n* = 4–6. Data is normalized against the average of the DMSO control. (**C**–**G**) qRT-PCR results showing the relative mRNA levels of downstream transcriptional targets of NRF2 activation in B16F10 treated for 24 h with control (DMSO), auranofin, TRi-1, or H_2_O_2,_ (median ± interquartile range). (**H**) The summary graph describing internal ROS levels assessed by a paired analysis of the control DHR signal vs. the indicated treatment. The experiment was performed on four different early passage (P12 or less) B16F10 cultures after 12 h of treatment. (**I**) Representative overlay histograms of B16F10 melanoma cells treated with the indicated reagent and concentration (colored) over the DMSO control (gray). Statistical significance for (**C**–**G**) was the Kruskal–Wallis non-parametric comparison test; *p* < 0.05 was considered significant and denoted as follows: * *p* < 0.05, ** *p* < 0.01, *** *p* < 0.001. For (**H**), significance was assessed by repeated measures one-way ANOVA and the two-stage Benjamini, Krieger, and Yekutieli multiple comparison with the adjusted *p*-value, *q* < 0.05, deemed as significant and denoted as: * *q* < 0.05.

Given the initial reports on the cytotoxic activity of TXNRD1 inhibitors, auranofin and TRi-1, on a range of cancer models through elevated ROS levels [12], we sought to test an underlying hypothesis in the field [8] (Figure 1A) and determine if similar results could be obtained with the B16F10 cell line (directly from the ATCC), a widely used cancer model for immunogenic metastatic melanoma. Using a modified version of the NCI-60 Human Tumor Cell Lines Screen protocol [37], we observed a reduction in cellular protein staining (Figure 1B) as seen through lower detection in normalized sulforhodamine B (SRB) absorption at 515 nm. Given that activation of NRF2 transcriptional targets by NRF2 is regulated by NRF2-KEAP1 interactions, which are disrupted by oxidation, we opted to look for increases in expression of transcriptional targets instead of direct changes in *Nrf2* expression. As expected, we also observed a compensatory increase in the relative expression of mRNAs in TXNRD1 inhibitor treated B16F10 cells from canonically activated genes downstream of NRF2 activation, such as *Txnrd1* (the gene encoding TXNRD1), NAD(P)H dehydrogenase quinone 1 (*Nqo1*), sulfiredoxin-1 (*Srxn*), heme oxygenase (*Hmox1*), and the cystine-glutamate antiporter (*Xct*), indicating activation of NRF2 (Figure 1C–G) [38,39]. Finally, we also observed a signal shift in the mean fluorescence intensity (MFI) of the redox-sensitive probe, dihydrorhodamine 123 (DHR), using flow cytometry, indicating elevated levels of intracellular ROS in cells treated with TXNRD1 inhibitors or H_2_O_2_ when compared to controls (Figure 1H,I).

Taken together, these data agree with initial reports studying auranofin and TRi-1 showing that at between 1 and 20 µM concentrations these drugs inhibit growth at comparable concentrations, as observed with other cancer models [12,40]. Thus, these data suggest that mice challenged with B16F10 may be amenable to auranofin or TRi-1 treatment.

### 3.2. Auranofin and TRi-1 Treatment Increased Tumor Burden in Lungs of Mice Challenged with B16F10

**Figure 2:**
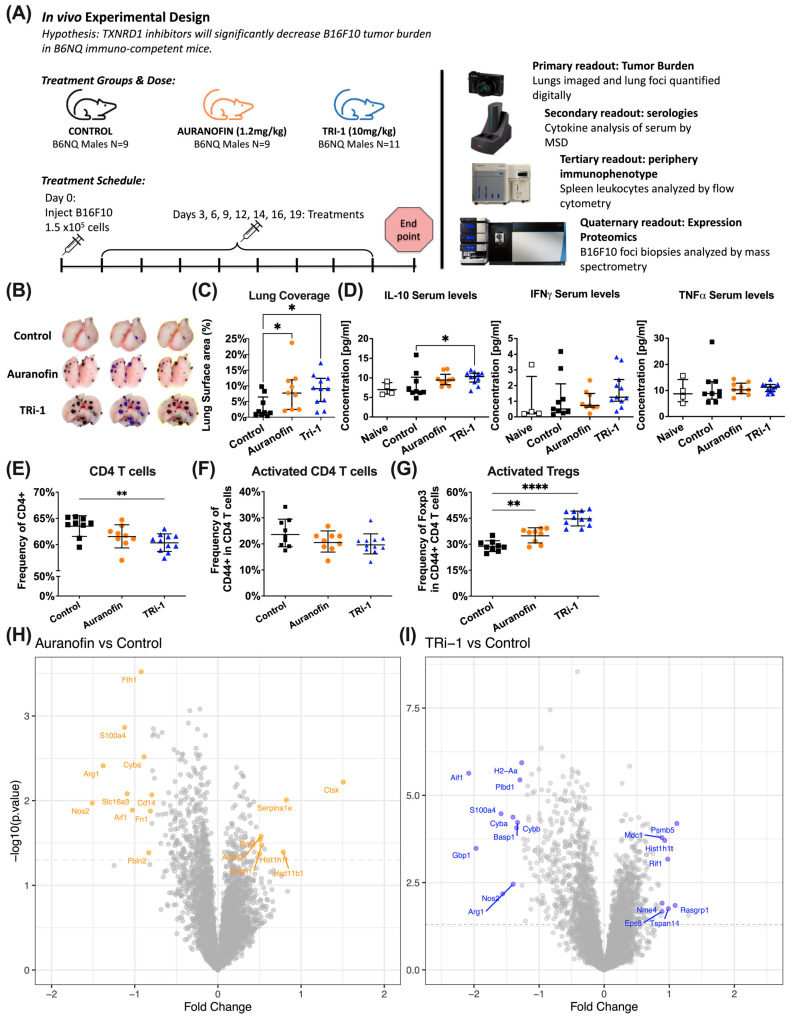
Experimental design and readouts for in vivo treatment of B16F10 with anti-cancer TXNRD1 inhibitors. (**A**) Experimental design and readouts: B6NQ mice 8–12 weeks old (males) were inoculated via tail vein with 1.5 × 10^5^ B16F10 cells at day 0. On day 3, they were treated I.P. with 100 µL of vehicle control (*n* = 9), or 1.2 mg/kg of auranofin (*n* = 9), or 10 mg/kg TRi-1 (*n* = 11) in approximately two-day intervals until day 20. (**B**) The representative photo shows the median tumor lung coverage measurement for each treatment group unmarked and with annotations. (**C**) The summary plot of the primary readout of tumor burden was assessed digitally and presented as the lung coverage of B16F10. (**D**) The serological cytokine levels from blood isolated at endpoint were analyzed by MSD and suggested elevated IL-10 levels in TRi-1 treated mice compared to the control treated. No differences were observed in serum levels of IFNɣ or TNFα. Plots display median, interquartile range. (**E**) Summary plot of leukocytes isolated from spleens at endpoint showing a decrease in CD4^+^ T cell frequency in the Lymphocyte > Singlets > live > TCRβ^+^ (Lineage: Lin) gate (median representative) in auranofin- and TRi-1 treated mice. (**F**) Summary plots show no difference was observed in the antigen-experienced CD4^+^ T cells through CD44^+^ staining between treatment groups. (**G**) Summary plots show significant FOXP3^+^ staining amongst the CD44^+^ CD4^+^ T cells in spleens taken from mice treated with auranofin and TRi-1. (**H**) The top differential expression proteomic analysis B16F10 lung foci biopsies from auranofin treated mice compared to control treated mice. (**I**) Similar representation of TRi-1 treated mice compared with control treated mice. Statistical significance for panels (**C**,**D**) was determined using the Kruskal–Wallis test followed by Dunn’s multiple comparisons (median ± interquartile range). For panels (**E**–**G**), significance was assessed using two-way ANOVA with Brown-Forsythe and Welch corrections followed by Dunnett’s multiple comparisons (geometric mean ± geometric SD). For panels (**H**,**I**), auranofin- or TRi-1–treated samples were compared to control-treated samples using a two-tailed unpaired *t*-test. A *p*-value < 0.05 was considered statistically significant and is denoted as follows: * *p* < 0.05, ** *p* < 0.01, **** *p* < 0.0001.

To determine if the in vivo B16F10 response to these inhibitors would align with their in vitro response, we designed in vivo treatment experiments using the B16F10 lung colonization model (Figure 2A). For auranofin, we wanted to treat mice equivalent to the indicated clinical dose of 6 mg [41] and thus used a calculated mouse dose, based on surface area conversion [42], of approximately 1.2 mg/kg. Given phase 1 pharmacokinetic studies of auranofin showing a blood steady-state concentration of gold between 1 and 1.5 µM [43], pharmacokinetic studies in mice support a mouse dose below 2 mg/kg to achieve a similar steady-state concentration [44]. For TRi-1, a novel TXNRD1 specific inhibitor, we aligned with initial studies, describing efficacy at a dose of 10 mg/kg [12]. Surprisingly, we found through two separate experiments that mice were not protected from metastasis in the lung after treatment with auranofin or TRi-1 but rather presented with greater numbers or coverage of B16F10 foci when compared to mice treated with the vehicle control (Figure 2B,C). We subsequently performed serologies and checked cytokine levels, including IL-10, IFNɣ, and TNFα, using an MSD detector (Figure 2D). We observed elevated IL-10 levels in mice treated with TRi-1. The elevation of IL-10 in mice treated with auranofin did not reach significance.

Given that auranofin is suggested to regulate T cell response in rheumatoid arthritis (RA) [4], we proceeded to immunophenotype these mice.

### 3.3. Tumor Bearing Mice Treated with Auranofin and TRi-1 Show Elevated Treg Formation When Compared to Control Treated Mice

**Figure A1:**
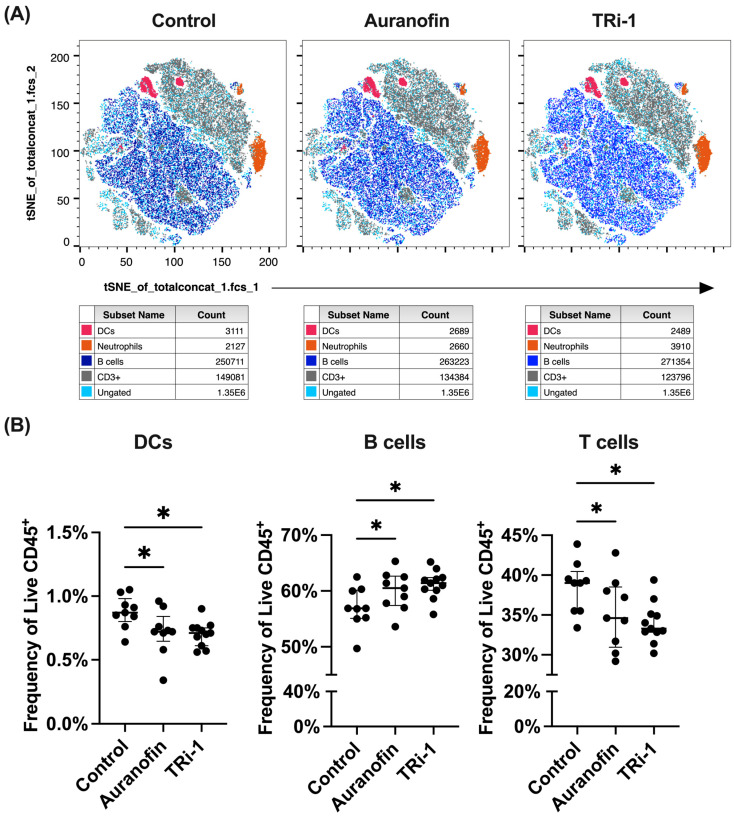
tSNE and immunophenotyping by flow cytometry. (**A**) Discovery via flow cytometry analysis using tSNE and (**B**) standard frequency analysis suggests meaningful differences in immune cell subsets from spleens of tumor bearing mice. A gating strategy (Lin^+^: Lymphocyte > singlets > live^+^CD45^+^) was employed to explore T cells (Lin^+^ > CD3^+^), B cells (Lin^+^ > CD3^−^ > CD19^+^), and DCs (Lin^+^: CD3^−^ > CD19^−^ >LY6G^−^ > CD11^+^MHCII^+^ (plots display median, interquartile range). Statistical significance was determined by a one-way ANOVA Kruskal–Wallis test with two-stage Benjamini, Krieger, and Yekutieli multiple comparisons. An adjusted *p*-value False discovery rate of (*q*) < 0.05 was deemed as a discovery and considered significant, indicated as follows: * *q* < 0.05.

**Figure A2:**
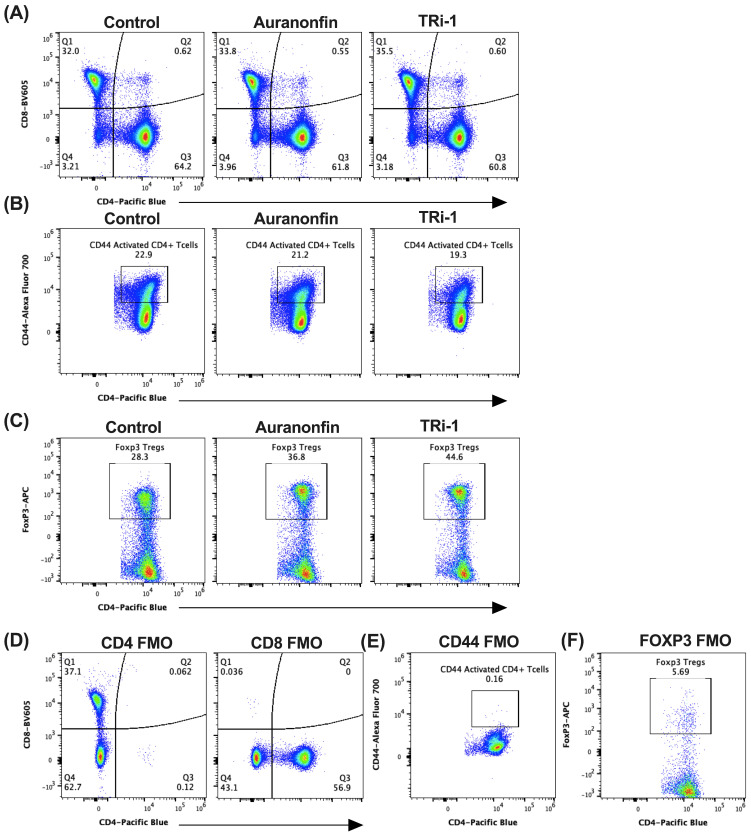
Representative flow cytometry and FMO controls for T cell immunophenotyping. (**A**) Flow cytometry plots of leukocytes isolated from spleens at endpoint show a decrease in CD4^+^ T cell frequency of auranofin- and TRi-1 treated mice in the Lymphocyte > Singlets > live > TCRβ^+^ (Lineage: Lin) gate (median representative). (**B**) Flow cytometry plots in the Lin > CD4^+^ gate (median representative) of antigen-experienced CD4^+^ T cells through CD44^+^ staining between treatment groups. (**C**) Flowcytometry plots in the Lin > CD4^+^ gate (median representative) of FOXP3^+^ staining amongst the CD44^+^ CD4^+^ T cells from spleens taken from mice treated with auranofin and TRi-1. (**D**–**F**) Disclose signal noise in the FMO controls used to establish positive signals and gates for the indicated antigen-fluorochrome. The data is presented in pseudocolor plots, a bivariate density plot displaying the relative population density where blue and green correspond to areas of lower cell density, red and orange for areas of high cell density, with yellow being mid-range.

By flow cytometric analysis of the spleen, we observed a relative decrease in the proportion of CD3^+^ T cells and DCs, as well as an increase in the proportion of B cells in mice treated with auranofin or TRi-1 (Figure A1). Looking further, we noted a marked decrease in total CD4^+^ T cells but an increase in regulatory T cells (Tregs), despite no change in the proportion of antigen-experienced CD44^+^ T cells from mice treated with auranofin or TRi-1 (Figure 2E–G and Figure A2).

These data suggest that TXNRD1 inhibitors suppress anti-tumor immunity through increased Treg expansion, possibly suppressing effector T cell activity. However, tumor burden positively correlates with the tumor’s immunosuppressive activity, and the contributions from the differences in B16F10 tumor burden among the treatment groups must also be acknowledged.

In summary, thus far the data only show a causative link between TXNRD1 inhibitor treatment and increased B16F10 tumor burden. The other observations are correlative but build support for investigating a causative link between TXNRD1 inhibitor treatment and Treg expansion.

### 3.4. Expression Proteomic and GO Enrichment Pathway Analysis Suggest Differences in Immune Response in Auranofin and TRi-1 Treated Mice

**Figure A3:**
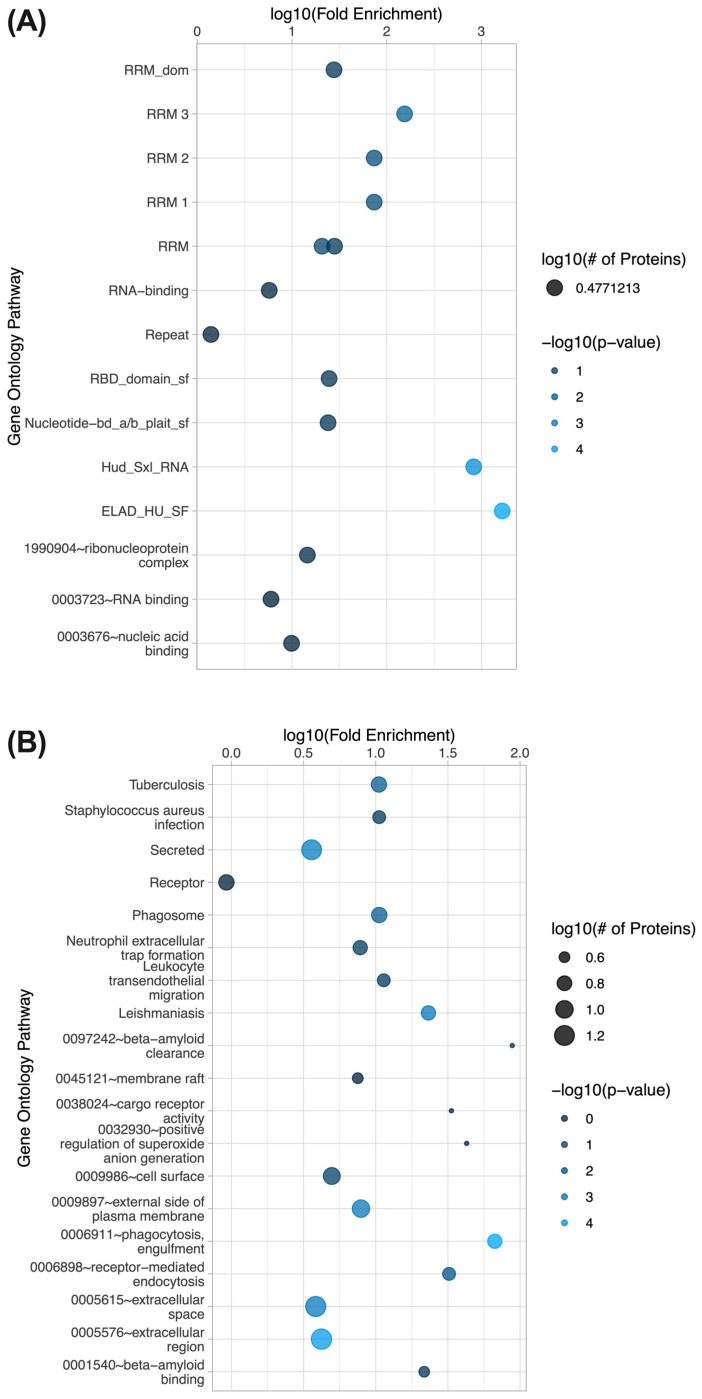
GO enrichment analysis of B16F10 lung foci between auranofin treated vs. control groups. (**A**) The top Gene Ontology (GO), including Biological Processes (BP), Molecular Functions (MF), and Cellular Components (CC), obtained from GO pathways enrichment pathways analysis using DAVID Function Clustering Annotation of all proteins differentially downregulated or (**B**) upregulated, were graphed accordingly with size related to the number of pathway-associated proteins. Statistical significance was assessed by Bonferroni correction with a *p*-value < 0.05 deemed as significant.

**Figure A4:**
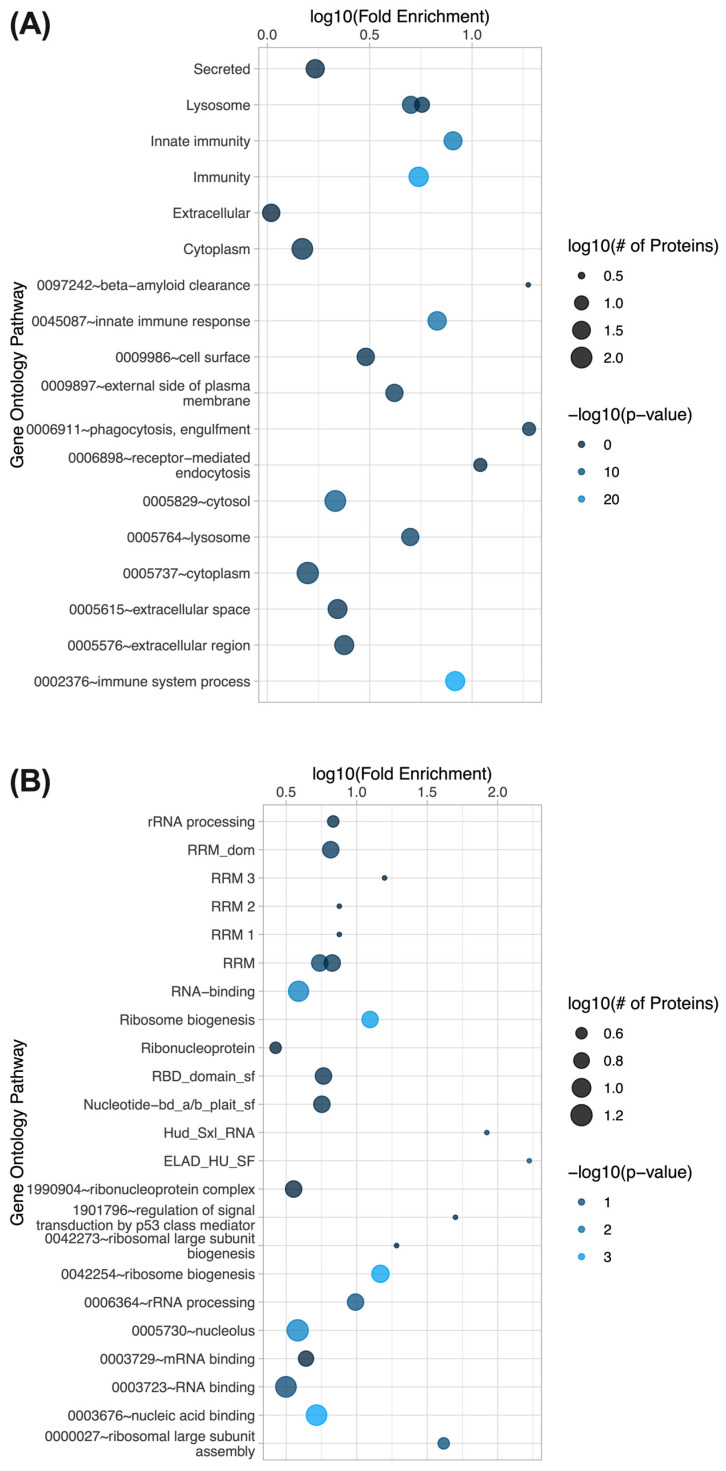
GO enrichment analysis of B16F10 lung foci between TRi-1 treated vs. control groups. (**A**) The top Gene Ontology (GO), including Biological Processes (BP), Molecular Functions (MF), and Cellular Components (CC), obtained from GO pathways enrichment pathways analysis using DAVID Function Clustering Annotation of all proteins differentially downregulated or (**B**) upregulated, were graphed accordingly with size related to the number of pathway-associated proteins. Statistical significance was assessed by Bonferroni correction with a *p*-value < 0.05 deemed as significant.

To gain further insight into the pathophysiological response to TXNRD1 inhibitor treatment in the tumor, we analyzed B16F10 lung foci and analyzed them for differences in protein expression (Figure 2H,I). The results of the top ten differentially expressed down-regulated proteins reveal coordinately lower levels of immune associated proteins such as CYBA, ARG1, NOS2, ARG1, and S100a4 in auranofin- and TRi-1 treated mice compared to controls. Furthermore, data obtained from GO enrichment pathways analysis using DAVID Function Clustering Annotation of all differentially expressed proteins also suggest differences in immune related pathways (Figure A3 and Figure A4). Notably, auranofin treatment did not provoke the strong downregulation of TXNL1 typically seen previously in proteomics studies using cancer cell line models [40,45,46], and neither auranofin nor TRi-1 seemed to have triggered clear upregulation of NRF2 targets in this data set.

Given that auranofin’s or TRi-1′s anti-tumoral efficacy had predominantly been evaluated in immunocompromised mice, we believed a direct assessment of auranofin’s and TRi-1′s effects on the immune response, free of the tumor’s influence, was warranted. Thus, we began to operate under a new working hypothesis:

**H1.**  *TXNRD1 inhibitors, such as auranofin, impairs anti-cancer immune responses in immunocompetent mice by promoting regulatory T cell expansion through elevating ROS levels.*

### 3.5. Naïve Murine Tregs Expand in Co-Cultured with Auranofin

**Figure 3:**
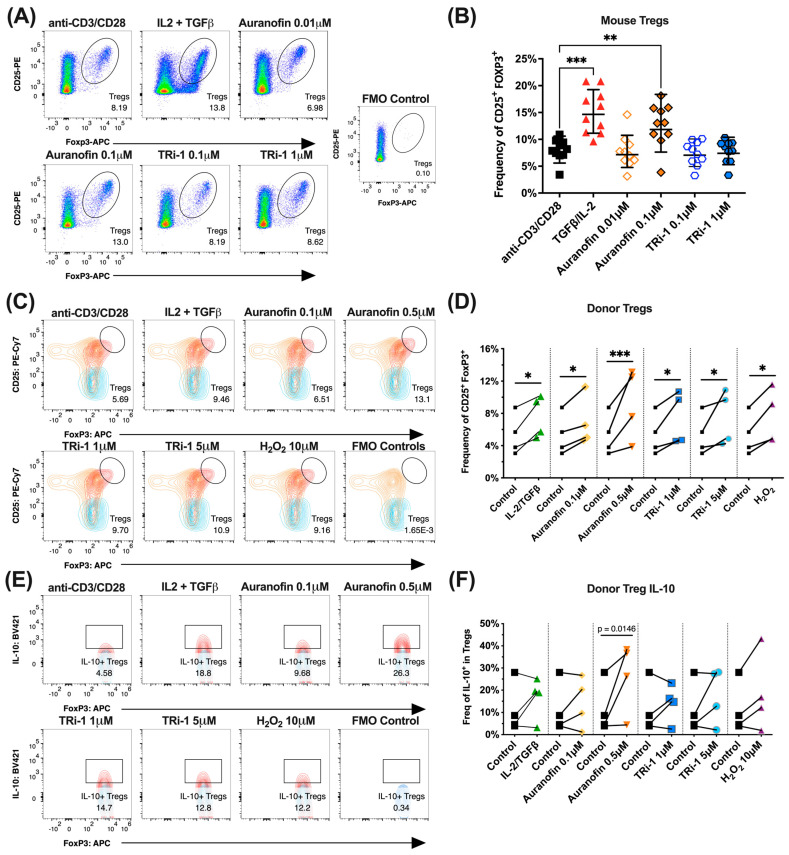
Murine and human Tregs expand in the presence of auranofin. (**A**) Median representative flow cytometry plots from lymph nodes isolated from *n* = 10 naïve mice and treated ex vivo with anti-CD3/CD28 alone or supplemented with IL-2 + TGFβ (positive control), or auranofin, or TRi-1 at indicated concentrations. The FOXP3 FMO control discloses signal noise. The data is presented in pseudocolor plots, a bivariate density plot displaying the relative population density where blue and green correspond to areas of lower cell density, red and orange for areas of high cell density, with yellow being mid-range. The oval gates identify CD25^+^ FOXP3^+^ Treg frequencies within the total CD4^+^ T cell population. (**B**) The summary plot shows a significantly expanded CD25^+^ FOXP3^+^ Treg frequency found in the total CD4^+^ gate (Live > TGFβ^+^ > CD4^+^) when treated with auranofin (geometric mean ± geometric SD). (**C**) Overlay flow cytometry contour plots of PBMCs from Donor 2 of 4 (red, average representative) present the frequency of CD25^+^ FOXP3^+^ Tregs (oval gate) in the CD4^+^ TGFβ ^+^ gate on top of the CD25 FMO control (blue) and the FOXP3 FMO control (orange). PBMC samples from healthy female donors (*n* = 4) were all treated with anti-CD3 and anti-CD28 either alone (control) or supplemented with IL-2 + TGFβ (positive control) or auranofin or TRi-1 or H_2_O_2_ at indicated concentrations. (**D**) The summary graph shows a paired analysis of control vs. treatment, with closed squares indicating anti-CD3/CD28 treatment. (**E**) Overlay contour plots of PBMCs from Donor 2 (average representative) present the frequency of IL-10^+^ (box gate) in the Treg gate (red) on top of the IL-10 FMO control (blue) and the FOXP3 FMO control (orange). PBMC samples from sex-matched healthy donors (*n* = 4) were stimulated with anti-CD3/CD28 alone (control) or supplemented with IL-2 + TGFβ (positive control) or auranofin or TRi-1 or H_2_O_2_ at indicated concentrations. (**F**) The summary graph shows a paired analysis of control vs. treatment from all donors, with closed squares indicating anti-CD3/CD28 treatment. Statistical significance for panel (**B**) was assessed using ANOVA with Geisser-Greenhouse correction followed by Holm–Sidak multiple comparisons, with the center bar representing geometric mean ± geometric SD. A *p*-value < 0.05 was considered significant and denoted as follows: ** *p* < 0.01, *** *p* < 0.001. For panels (**D**,**F**), significance was determined by repeated-measures one-way ANOVA with either two-stage Benjamini, Krieger, and Yekutieli multiple comparisons or Dunnett’s test. An adjusted *p*-value (*q*) < 0.05 was deemed significant and indicated as follows: * *q* < 0.05, *** *q* < 0.001. Note the individual *p*-value for auranofin 0.5 µM was <0.05.

Having confirmed the in vitro activation of NRF2 by auranofin and TRi-1 and given the observed expansion of peripheral Tregs, we were keen to assess basic T-cell responses in the presence of TXNRD1 inhibitors [12]. We also did not know if our observations were mediated by the inhibitors’ effects on T cells or antigen-presenting cells (APCs) or by some combination of both. Thus, we opted not to test sorted cells. To validate if auranofin and TRi-1 promote Treg formation, we isolated lymph node cells from naïve mice and stimulated them for 3–4 days with anti-CD3/CD28. We observed an increase in the frequency of CD25^+^ FOXP3^+^ Tregs cultured in the presence of auranofin when compared to the control, indicating that auranofin can promote Treg expansion upon T cell receptor (TCR) stimulation with anti-CD3/CD28 and was comparable to Treg expansion obtained through treatment with IL-2 and TGFβ (Figure 3A,B).

### 3.6. Healthy Human Donor Tregs Expand in Co-Culture with Auranofin or TRi-1

**Figure 4:**
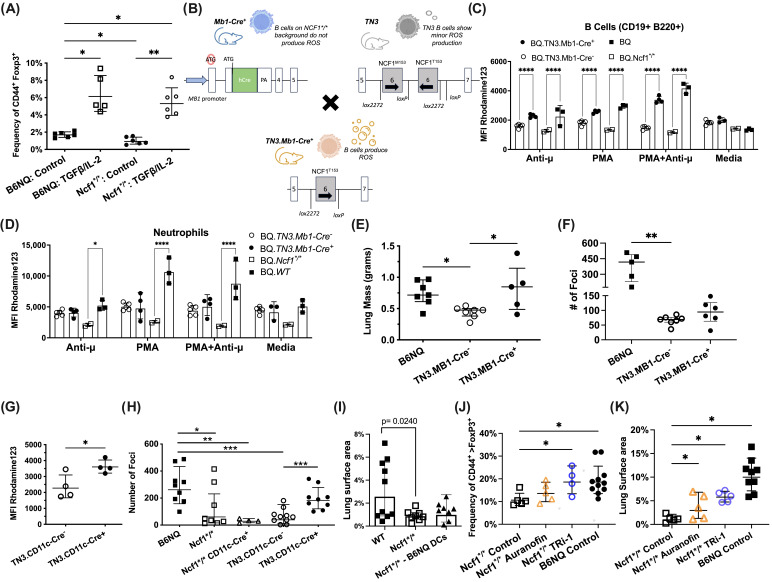
B cell or DC restricted NCF1-NOX2-ROS promotes B16F10 tumor progression, but auranofin induced ROS can expand Tregs and B16F10 tumors without NOX2. (**A**) Flow cytometry analysis compares ex vivo Treg expansion in wild-type B6NQ and mutant Ncf1*^/^* strains when stimulated with anti-CD3ε/CD28 (*n* = 5–6) and when polarized further with the addition of TGFβ and IL-2. The frequency of Tregs (CD44^+^ FOXP3^+^) in total live > TGFβ > CD4^+^ cells (geometric mean ± geometric SD). (**B**) Schematic depicting the generation of TN3.MB1-cre^+^ mice with B cell specific NOX2-NCF1-ROS production. The schematic is analogous to the production of TN3.CD11c-Cre^+^ mice with DC specific ROS production. (**C**) Flow cytometry analysis of the DHR assay for oxidative burst on naïve spleens indicated elevated ROS levels in B220^+^ CD19^+^ B cells (geometric mean ± geometric SD), and (**D**) neutrophils from TN3.Mb1-Cre^+^ mice compared to TN3.Mb1-Cre^−^ mice (geometric mean ± geometric SD). (**E**) B16F10 lung tumor burden in advanced stage disease (Day 28, *n* = 5–7), precluded foci counting or lung area assessment and was determined by lung mass, showing significantly greater tumor burden in ROS producing strains B6NQ and TN3.Mb1-Cre^+^ (median ± interquartile range). (**F**) Separately, B16F10 lung tumor burden at Day 21 (*n* = 5–7) was determined by the number of lung foci, showing augmented tumor growth in TN3.Mb1-Cre^+^ mice compared to littermate controls TN3.Mb1-Cre^−^ (median ± interquartile range). (**G**) Flow cytometry analysis of the DHR assay for oxidative burst on naïve spleens indicated elevated ROS levels in CD45^+^ CD11c^+^ cells from *TN3.CD11c-Cre*^+^ mice compared to *TN3.CD11c-Cre*^−^ mice (*n* = 4), (geometric mean ± geometric SD). (**H**) The number of B16F10 lung foci was significantly greater in ROS producing strains B6NQ and *TN3.CD11c-Cre*^+^, and evidence for DC specific ROS being sufficient to drive the phenotype (*n* = 4–10), (geometric mean ± geometric SD). (**I**) B16F10 lung area coverage was not significantly different in Ncf1*^/^* mice receiving adoptive transfer of B6NQ BMDCs compared to B6NQ mice as opposed to Ncf1*^/^* vs. B6NQ (*n* = 8–9), (geometric mean ± geometric SD). (**J**) Flow cytometry analysis of lung homogenate from *Ncf1* mutant B16F10 tumor bearing mice treated with TXNRD1 inhibitors shows elevated levels of FOXP3^+^ regulatory T cells. (Gating strategy: Lymphocytes > singlets > live > TCRB^+^ > CD8^−^) The frequency of Tregs (CD44^+^ > FOXP3^+^) in total live > TCRβ > CD4^+^ cells, (geometric mean ± geometric SD). (**K**) B16F10 tumor burden assessed by lung surface area coverage shows greater tumor growth in auranofin- and TRi-1 treated mice than in the control treated, mirroring the Treg frequencies, (geometric mean ± geometric SD). Statistical significance ± SE was determined as follows: one-way ANOVA using Brown-Forsythe and Welch corrections with Dunnett’s multiple comparisons (**A**); two-way ANOVA (**C**,**D**); one-way ANOVA with Kruskal–Wallis test followed by Dunn’s multiple comparisons (**E**,**F**); two-tailed *t*-test (**G**); one-way ANOVA with Mann–Whitney test for comparisons between TN3.CD11c-CRE^+^ and TN3.CD11c-CRE^−^ (**H**); and standard ANOVA for panels (**I**–**K**). A *p*-value < 0.05 was considered statistically significant and is denoted as: * *p* < 0.05, ** *p* < 0.01, *** *p* < 0.001, **** *p* < 0.0001.

To test if auranofin and TRi-1 could promote Treg expansion in primary human T cells, we isolated PBMCs from four healthy donors and stimulated them for 3 days with anti-CD3ε/CD28 (*n* = 4). We observed an increase in the frequency of CD25^+^ FOXP3^+^ expressing T cells cultured in the presence of auranofin or TRi-1 when compared to controls. These data provide preliminary evidence indicating that TXNRD1 inhibitors may promote Treg expansion in humans as well as in mice. Results from both human and mouse samples also suggest that auranofin-induced Treg expansion can be achieved independent of tumor-specific antigens or the tumor microenvironment (Figure 3C,D). Moreover, upon treatment with auranofin, these Tregs also produced elevated levels of IL-10 (Figure 3E,F). Given that the mechanism of action for TXNRD1 inhibitors involves an accumulation of intracellular ROS, including H_2_O_2_, we also tested H_2_O_2_ at an elevated, physiologically relevant level [47] of 10 µM and observed a significant Treg expansion, supporting previous observations of ROS-mediated immune regulation [48]. Finally, we and others observed suppressed murine Treg expansion in Ncf1*^/^* mutant chronic granulomatous disease (CGD) mice with anti-CD3ε/CD28 stimulation (Figure 4A), which was reestablished during Treg polarization with robust TGFβ signaling. This suggested that biological producers of H_2_O_2_ such as immune cells expressing NCF1/NOX2 may also play a direct role in Treg expansion, impacting anti-tumor immunity.

### 3.7. B Cell Specific NOX-2 ROS Augments B16F10 Tumor Burden

Given that we also noted significant frequency differences in B cells and dendritic cells from spleens of auranofin and TRi-1 treated mice when compared to control treated mice (Figure A2), we were keen to observe the possible contribution of these immune cell subtypes towards B16F10 disease progression. We believed systemic treatment with auranofin and TRi-1 likely affects B cells and DCs, possibly activating NRF2, and up-regulating their immunosuppressive activity. We previously observed that NOX2-ROS promotes the growth of implanted tumors using Ncf1*^/^* mutant mice [26]. In this study, we decided to investigate the contribution of B cell derived NOX2-NCF1-ROS towards B16F10 tumor progression, using a conditional knock-in on our Ncf1*^/^*mutant stain.

We crossed *TN3* mice, as previously described [36,49], lacking functional NOX2-ROS production with Ncf1*^/^*mutant mice carrying a *Cre*-recombinase allele controlled by the *MB1 (CD79a)* locus (*Mb1-Cre*) (Figure 4B). The resulting F1 litter was *TN3.Mb1-Cre*^+^, knocking in a single functioning *Ncf1* allele. By flow cytometry, we confirmed that *TN3.MB1-Cre*^+^ B cells, as defined as CD19^+^ B220^+^, had restored NOX2-ROS production, when compared with their *TN3.MB1-Cre*^−^ littermate controls (Figure 4C). We also confirmed that both *TN3.MB1-Cre^+^* and *TN3.MB1-Cre*^−^ were otherwise NOX2-ROS deficient with a readout on LY6G^+^ neutrophils, as similarly observed in the Ncf1*^/^* strain when compared to the wild-type B6NQ strain (Figure 4D).

Mindful that Ncf1*^/^* mice have stunted tumor progression when compared to B6NQ mice, we extended our disease model from 10 to 21 days to 28 days. This resulted in advanced B16F10 disease progression, which precluded tumor foci counting or digital surface area assessment. Therefore, using lung mass, we observed a greater tumor burden in *TN3.Mb1-Cre^+^* when compared to *TN3.Mb1-Cre*^−^ littermate controls (Figure 4E). Upon re-evaluation in a 21-day model, we observed a more modest increase in tumor burden in *TN3.MB1-Cre*^+^, when compared to littermate controls (Figure 4F).

### 3.8. DC Specific NOX-2 ROS Augments B16F10 Tumor Burden

To obtain a DC NOX2-ROS specific strain, we similarly produced *TN3.CD11c-Cre*^+^ mice and observed restored NOX2-ROS production in the knock-in mice, compared with *TN3.CD11c-Cre*^−^ littermate controls (Figure 4G). In two separate experiments, *TN3.CD11c-Cre*^+^ mice had an increased tumor burden when compared to their littermate controls (Figure 4H). We noted a greater pro-tumoral effect in DC restricted NOX2-ROS at 21 days than those from experiments focused on B cell restricted NOX2-ROS in the *TN3.MB1-Cre*^+^ strain (Figure 3F).

We tested the adoptive transfer of bone marrow derived DCs from B6NQ into Ncf1*^/^* mutant hosts bearing B16F10 tumors and observed a modest result showing a greater tumor burden that did not reach statistical significance (Figure 4I).

### 3.9. TXNRD1 Inhibitors Expand Tregs and Augment B16F10 Tumors in Ncf1*^/^* Mice

The NCF1^m1j^ mutation in the Ncf1*^/^* strain leads to a decreased capacity to produce ROS by the NOX2 complex but does not diminish other sources of ROS, such as mitochondrial ROS production [34]. To determine the effect of treatment without an interaction with the NOX2 complex, we therefore used Ncf1*^/^* mice lacking NOX2-derived ROS. We observed increased frequencies of Tregs from the lungs of auranofin and TRi-1 treated Ncf1*^/^* mice vs. controls as well as significantly augmented B16F10 tumor burden (Figure 4K). Notably, these data show auranofin and TRi-1 treated Ncf1*^/^* mice present with Treg frequencies and tumor burden approaching in similarity to that of control treated wild type mice (Figure 4J). Furthermore, when considered together with the augmented tumor growth from B cell and DC restricted NOX2-ROS, these data suggest that auranofin-induced ROS likely stimulate Treg polarization and expansion contemporaneously with T cell priming by APCs.

### 3.10. Txnrd1^fl/fl^ CD4-Cre Mice Upregulate NRF2 Target Genes and Reduce B16F10 Tumor Burden

**Figure 5:**
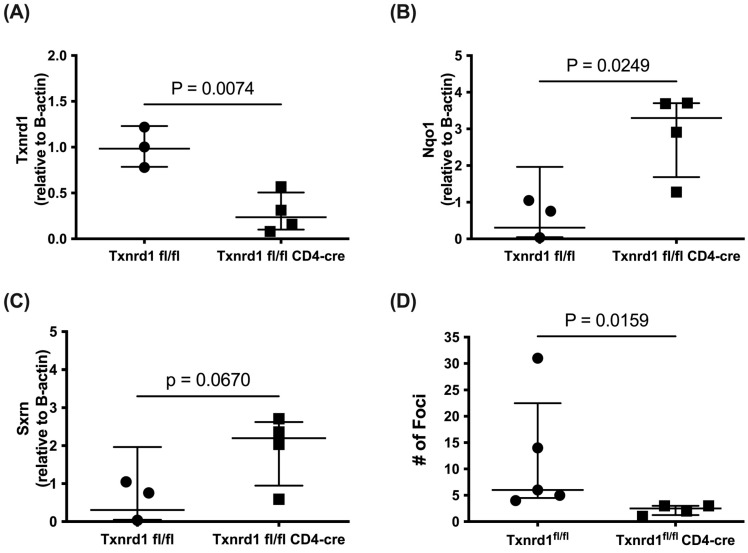
CD4^+^ T cells from Txnrd1^fl/fl^ CD4-Cre mice show reduced *Txnrd1* expression, increased Nrf2 activation, and stunted B16F10 tumor progression. (**A**) qRT-PCR data from MACs sorted CD4 T cells showing relative expression of *Txnrd1*, (**B**) *Nqo1*, and (**C**) *Sxrn* mRNA levels in Txnrd1^fl/fl^ (*n* = 3) and Txnrd1^fl/fl^ CD4-Cre (*n* = 3–4), (geometric mean ± geometric SD). (**D**) Lung foci of B16F10 in Txnrd1^fl/fl^ (*n* = 5) and Txnrd1^fl/fl^ CD4-Cre (*n* = 4), (median ± interquartile range). Statistical significance was determined by unpaired *t* tests (**A**–**C**), and a Mann–Whitney test with *p* < 0.05 was considered significant.

Thus far the data has shown causal links (1) between TXNRD1 inhibitor treatment and increased B16F10 tumor burden, (2) between TXNRD1 inhibitor treatment and increased Treg expansion in mice and human cultures, (3) between a mechanistic byproduct of TXNRD1 inhibitor treatment (H_2_O_2_) and increased Treg expansion, and (4) between biological producers of H_2_O_2_, (B cells and DCs) and increased B16F10 tumor burden. To evaluate the intrinsic role of TXNRD1 blockade in T cells, we created conditional knockouts using Txnrd1^fl/fl^ CD4-cre mice. We confirmed the loss of *Txnrd1* expression in T cells with RT-PCR analysis on MACS sorted CD4^+^ naïve T cells (Figure 5A). We also observed compensatory upregulation of the NRF2 target gene *Nqo1*, with only a marginal increase in *Sxrn*, suggesting constitutively activated NRF2 signaling from *Txnrd1* ablation (Figure 5B,C).

A preliminary evaluation of B16F10 tumor progression in Txnrd1^fl/fl^ CD4-cre mice showed reduced tumor burden compared to their littermate controls (Figure 5D). At first glance, these data suggested that *Txnrd1* blockade in T cells improves antitumor immunity. However, studies from Muri et al. suggest that *Txnrd1* blockade in T cells with a similarly created strain of Txnrd1^fl/fl^ CD4-cre severely impacts their ability to proliferate and mount an effective immune response against viral infections [50]. As we did not perform functional assays on T cells from this strain, our ability to interpret the results from this animal study is limited. What is clear is that the Txnrd1^fl/fl^ CD4-cre mice do not mirror TXNRD1 inhibitor treatment, as T cells treated with auranofin or TRi-1 still proliferate.

### 3.11. CD4^+^ T Cells from TXNRD1^fl/fl^ CD4-Cre Mice Express Less FOXP3 and GITR

**Figure 6:**
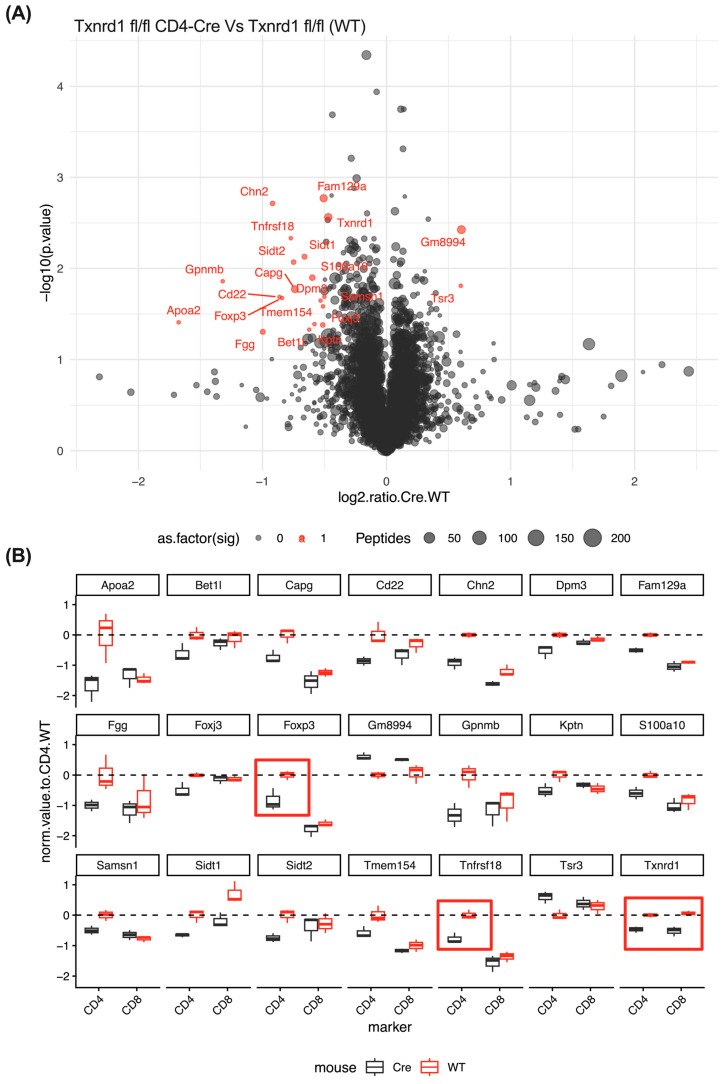
Selected proteins levels are significantly different in Txnrd1^fl/fl^ CD4-Cre vs. Txnrd1^fl/fl^. (**A**) A volcano plot showing proteins from FACs-sorted CD4 T cells from Txnrd1^fl/fl^ CD4-Cre vs. Txnrd1^fl/fl^ (denoted as WT), with curated proteins highlighted in red. (**B**) Box plots with red boxes denoting reduced protein levels of TXNRD1 and essential Treg biomarkers FOXP3 and TNFRSF18 (GITR) in CD4^+^ T cells from Txnrd1^fl/fl^ CD4-Cre vs. Txnrd1^fl/fl^. Data was normalized to protein levels of Txnrd1^fl/fl^ CD4^+^ T cells. Statistical significance was determined by Welch’s two sample *t* test.

Finally, to gain further insight into the intrinsic role of TXNRD1 blockade in T cells, we FACS sorted CD4^+^ and CD8^+^ naïve T cells from TXNRD1^fl/fl^ CD4-Cre mice and performed expression proteomic analysis (Figure 6A,B). When comparing TXNRD1^fl/fl^ CD4-Cre^+^ T cells to Cre^−^ T cells, we confirmed reduced TXNRD1 protein in both CD4^+^ and CD8^+^ T cells. We also noticed a significant reduction in two constitutively expressed Treg proteins, namely FOXP3 and TNFRSF18 (GITR). These data are preliminary but suggest that TXNRD1^fl/fl^ CD4-Cre^+^ mice may be predisposed to lower levels of regulatory T cells at homeostatic conditions. FOXP3 and TNFRSF18 (GITR) are essential to Treg functionality, highlighting TXNRD1′s possible influence on Treg portions during development.

## 4. Discussion

This study reports, for the first time, auranofin-induced regulatory T cell expansion. It provides a rationale for the divergent anti-tumoral responses to auranofin treatment observed in immunocompetent [13,24] versus immunocompromised [12] cancer models. Tregs are known to play a crucial role in cancer immune evasion, a hallmark of cancer [51]. We demonstrate a direct causal relationship between treatment with the TXNRD1 inhibitors auranofin and TRi-1 and enhanced in vivo B16F10 tumor progression using the syngeneic lung colonization model. B16F10 is extensively studied, and its poor immunogenicity and growth are shown to be dependent upon Tregs [52]. With this in mind, we also link our correlative observation of in vivo Treg expansion in B16F10 bearing mice, undergoing TXNRD1 inhibitor treatment, with causal experiments validating direct Treg expansion from said treatment with ex vivo cultures of naïve murine lymph nodes. These results demonstrate that tumor antigens, type of tumor, or overall burden are not essential for this activity, though they may have an influence. Moreover, we replicate TXNRD1 inhibitor-induced Treg expansion in PBMC cultures from healthy human donors to show potential clinical relevance. To show direct involvement of the underlying mechanism of REDOX dysregulation from TXNRD1 blockade, we confirm that TXNRD1 inhibitors elevate ROS and activate NRF2 and show that treatment with auranofin or hydrogen peroxide—also a byproduct of NCF1-NOX2 in APCs—induces Treg polarization and expansion that is comparable to IL-2 + TGFβ treatment. To demonstrate biological relevance and tie our observations with immunocompetent models, we focus on immune mediators of ROS production and use conditional NCF1 knock-in models to show that elevated ROS levels from pharmacological TXNRD1 blockade or from APCs expressing NOX2 are comparable enhancers of B16F10 tumor progression. These data suggest that auranofin-induced ROS is sufficient to drive Treg expansion and foster tumor growth independently of NCF1-NOX2-ROS. Together with the ex vivo Treg expansion assays, conditional NCF1 knock-in models show that Treg expansion from elevated ROS levels must occur proximal to and concurrent with T cell priming by APCs. Thus, the role of ROS, and to some extent NRF2 signaling, in shaping anti-tumoral immunity may explain inconsistent responses to auranofin in different models and guide more effective use of TXNRD1 inhibitors in cancer therapy.

While there are many limitations to this study, the most obvious limitation comes from what we do not show. We do not show a direct line from TXNRD1 inhibitor treatment to in vivo Treg expansion. We do not show that these Tregs then lead to augmented B16F10 tumor burden. Instead, our interpretation of the results relies heavily on correlating several causal links that we demonstrate, namely between treatment and enhanced tumor burden, treatment and expanded Tregs, ROS and expanded Tregs, and finally ROS and enhanced tumor burden. We do not demonstrate a continuous causal chain to show TXNRD1 inhibitors elevate APC ROS levels that in turn drive Treg expansion and immune suppression, leading to augmented tumor growth. We rely on a strong association between our experiments and on several important causal and correlative observations in the literature, which we explain below, that coalesce and align with our model.

Auranofin effectively reduces growth in several cancer models, in large part, by inhibiting innate ROS scavenging enzymes in malignant tissue with pre-existing elevated levels of ROS and NRF2 activation. However, increased ROS activity on leukocytes can result in immunosuppression [53,54,55]. ROS production in antigen presenting cells can lead to Treg expansion, as demonstrated in studies of immune regulation of autoimmune diseases [36,48]. Auranofin-induced ROS may work similarly. ROS in antigen presenting cells could both operate intrinsically, affecting the function of ROS producing cells, and extrinsically, operating as an immunological transmitter affecting interacting T cells [56]. Alternatively, activation of NRF2 may lead to a therapeutic anti-inflammatory response, a mechanism already leveraged in clinical practice with approved treatments such as dimethyl fumarate and omaveloxolone [57,58,59,60,61,62]. In addition, Treg expansion is linked to the upregulation of cystine/glutamate transporter *Slc7a11* and the coding protein XCT, which are downstream of NRF2 activation [63]. Moreover, suppression of XCT increases anti-tumor immunity [64,65]. Thus, we believe the expansion of auranofin-induced Tregs results, in part, from elevated levels of ROS and from NRF2 activation.

Notably, the role of NRF2 in immunity is still controversial. Some report observations of enhanced immune suppression from the loss of NRF2 signaling in Tregs [66], and others report that NRF2 signaling leads to immune activation and loss of tolerance [67]. Ultimately, however, no clinically available therapies currently rely on NRF2 inhibition as their primary mechanism of action.

Consider N-acetyl cysteine (NAC) as an example of our efforts to understand the relationship between ROS and NRF2 in immune response. NAC is clinically available and shown to inhibit NRF2 indirectly through robust glutathione production. While NAC is recognized as an antioxidant that suppresses Nrf2 activation, NAC has a limited direct reductive capacity [68] but relies in part on increased XCT activity, which we point out is important for maintaining Treg functionality and is also an Nrf2 transcriptional target. Expressly, NAC is recognized for elevating cysteine levels for glutathione production. However, NAC may also elevate levels of the short chain fatty acid, acetate, a metabolite shown to promote Treg expansion [69], supplied in molecular equivalents to NAC treatment through the deacetylation of acetyl cysteine in steps prior to glutathione synthesis [70]. Furthermore, increased XCT activity could also lead to decreased glutamate levels [71], and glutamate depletion has been shown to promote Treg expansion [72]. In short, NAC is known to expand Tregs both on the bench and in the clinic [73], this expansion likely occurs through two known pathways, namely, increases in short chain fatty acid levels and reductions in intercellular glutamate levels. Thus, NAC may mitigate ROS induced Treg expansion by increasing reductive equivalents through elevated glutathione production, but it ultimately promotes Treg expansion, and may be the reason it, like auranofin, also promotes B16F10 tumor growth in vivo [74].

We suspect auranofin-induced Treg expansion likely works through elevating ROS levels, which could not only activate NRF2 but may also separately augment TGFβ signaling, whose activation is known to be ROS-dependent [75,76]. Studies show treatment with gold salts enhances TGFβ signaling, augments Treg frequencies, and increases immune suppression [77]. Our results go further and directly tie auranofin treatment to Treg expansion. Given that gold salts, including auranofin, inhibit the selenocysteine active sites of antioxidant enzymes [5,7,78], epidemiological observations in selenium-deficient populations of Kashin–Beck disease and Chagas disease could provide insight, as they report elevated levels of both ROS and TGFβ [79,80]. Increased TGFβ levels are also found in selenium deficient colitis mouse models [81]. Furthermore, investigations on animal models with myeloid specific loss of selenoproteins report elevated ROS levels and greater tumor burden compared to controls [82], demonstrating directly what our experiments imply separately. Thus, auranofin’s inhibition of selenoproteins augments ROS levels and may act through elevating APC ROS levels as well as through increased TGFβ signaling, providing additional possible mechanisms of action for auranofin-induced Treg expansion.

We previously implicated NCF1-NOX2-ROS in anti-tumor immunity [26], consistent with findings in CGD patients who exhibit reduced ROS production, TGFβ levels, and Treg frequencies [48,83]. While loss-of-function NOX2 mutations in mouse cancer models often reduce tumor burden [49,84,85], notable exceptions exist. Transgenic and chemically induced models such as TRAMP and MCA [26,86], which produce high TGFβ levels [87], are not susceptible to anti-tumor immunity from NCF1-NOX2-ROS deficiency but, like B16F10 [88], are sensitive to anti-TGFβ and anti-Treg treatment [87,89,90].

The role of TGFβ is of further interest given data suggesting myeloid specific TGFβ signaling has a crucial role in metastasis [91], and those that show B cell specific TGFβ can induce Tregs [92]. Findings suggesting the critical role of NCF1-ROS in TGFβ-mediated Treg suppression of effector T cells are of particular interest [93]. While these studies highlight TGFβ signaling in immune cell interactions and immune response, TGFβ’s ubiquitous presence in vivo, supplied principally from platelets [94], and a pre-existing supply on the extracellular membrane of Tregs [95], are contributing factors and may confound our understanding regarding the extent to which an immune cell impacts immunity through TGFβ production [96,97,98].

Thus, we focus on B-cell specific and DC-specific ROS designed to understand where auranofin-induced ROS might critically impact anti-tumor immunity, given that Treg polarization or expansion must occur during T cell priming or subsequent activation. B cells can regulate immune response against cancer, with therapeutic anti-tumoral efficacy dependent upon the depth of B cell depletion [99,100,101]. Our data suggest targeting B-cell specific ROS may be sufficient in regulating B16F10 anti-tumor immunity in NCF1-deficient mice. Alternatively, our data also suggests the same for DC-specific ROS, complementing reports of DC-ROS induction via dimethyl fumarate-treated bone marrow-derived DCs adoptively transferred to regulate autoimmunity in an experimental autoimmune encephalomyelitis (EAE) mouse model [29].

## 5. Conclusions

This study provides the first report of auranofin-induced regulatory T cell expansion. We suggest a mechanism through elevated ROS levels and NRF2 activation that is concurrent with T cell priming by antigen presenting cells. However, auranofin may also promote Treg expansion through related molecular pathways involving ROS-enhanced TGFβ signaling and other immune cell mediators, though these remain to be confirmed. This study has several additional limitations that temper the impact of the results, where we imply that TXNRD1 inhibitors foster augmented B16F10 tumors through ROS-induced Treg expansion and subsequent immune suppression. We describe a small but significant difference in tumor burden, which could have benefitted from larger sample sizes for greater statistical power. We also do not directly test the suppressive function of Tregs expanded in the presence of TXNRD1 inhibitors beyond a cursory look at IL-10 production. Instead, we rely, in part, on B16F10′s known susceptibility to changes in Treg frequencies [102], which is widely accepted, and for which is the foundation of an established in vivo regulatory T cell suppression assay involving Tregs and B16F10 [103]. Our study is also limited by having tested only a single tumor model. Finally, while we tested both males and females agnostically in our studies based on availability for littermate controls, we did not ensure that we tested both sexes for each study. Despite these limitations, on balance our data demonstrate several causative relationships between ROS, Tregs, and tumor burden, including a first described causal relationship between TXNRD1 inhibitors like auranofin and Treg expansion.

Given that previous work has established the causal relationship between Tregs and the suppression of anti-tumor immunity, we believe future studies should begin by using these models. Recall that Turk et al. use Rag KO mice to show several adoptive transfer conditions including how a co-injection of Tregs with CD8^+^ T cells suppresses the CD8^+^ T cells’ antitumor immunity and augments B16 tumor growth when compared to CD8^+^ T cells alone [52]. In addition, studies by Noyes et al. show augmented tumor burden in the B16F10 model, in C57BL6J mice, from a targeted tamoxifen-induced depletion of FOXP3 Tregs [102]. These mice have the advantage of developing naturally, with a fully functioning immune system, and have a capability for targeted depletion of Tregs under experimental conditions. While there are other examples in the literature, these two studies cover the basics of interventional experiments involving Tregs in the B16F10 model, that is, if you add Tregs, then tumors increase, and if you deplete Tregs, then tumors decrease. These studies, among others, in our view have established the causal relationship between Tregs and the suppression of anti-tumor immunity of B16F10.

A major advantage of our study over initial treatment studies is that we leverage these findings by using the B16F10 in C57BL6 mice to demonstrate TXNRD1 inhibitors are sufficient in augmenting Treg frequencies and tumor burden without other interventions.

The next steps in investigating the immunological effects of TXNRD1 inhibitor treatment could involve *Rag KO* and *Foxp3^ERT2^* models. Moreover, small molecules are notoriously promiscuous, and additional studies establishing a stronger link between TXNRD1 inhibition and Treg expansion should focus on using more precise genetic models/tools than those used in our study. Studies focused on antigen-specific responses would also be useful. Finally, an extension of our findings to studying other cancer models and autoimmunity models would demonstrate broad application.

In summary, our study highlights a critical immunological aspect of auranofin’s mode of action. Our findings provide insight for understanding the differential anti-tumoral responses to auranofin observed in various models and for refining therapeutic strategies involving TXNRD1 inhibitors, including strategies for possible combinational therapies.

## Data Availability

The mass spectrometry proteomics data have been deposited to ProteomeXchange Consortium (http://proteomecentral.proteomexchange.org) via the PRIDE partner repository with data set identifier PXD060423 (B16F10 in vivo treated tumors) and PXD069997 (FACS sorted naïve CD4^+^ & CD8^+^ T cells from Txnrd1^fl/fl^CD4-cre ± mice [104]. For all other data, inquiries can be directed to the corresponding author.

## Abbreviations

The following abbreviations are used in this manuscript:

TXNRD1	Thioredoxin reductase 1 (TXNRD1)
Treg	Regulatory T cell
Ncf1	neutrophil cytosolic factor 1
APC	antigen-presenting cells
NOX2	NADPH oxidase 2

## Appendix A

**Table A1:** Reagents.

Reagent or Resource	Source	Cat Number	Dilution/ Concentration	RRID
Auranofin	R&D Systems	4600/50		
TRi-1	Arner Lab			
Cell Dissociation Buffer, enzyme-free, PBS	Gibco	13151014		
B16F10	ATCC	CRL-6475		CVCL_0159
Ionomycin	ThermoFisher	I24222	1 μg/mL	
BFA	ThermoFisher	B7450	5 μg/mL	
PMA	Sigma-Aldrich	CAS#: 16561-29-8 Cat. P8139	100 ng/mL	
dimethylsulfoxide (DMSO)	Sigma-Aldrich	CAS# 67-68-5		
DHR	ThermoFisher	D23806	3 μM	
Fixable Live/Dead NIR	ThermoFisher	L10119	1:1000	
BD cytofix/cytoperm solution	BD Biosciences	554714		AB_2869008
iScript™ cDNA Synthesis Kit	Bio-Rad	1708891		
Sulforhodamine B sodium salt	Sigma-Aldrich	S1402-1G		
QIAshredder (50)	Qiagen	79654		
RNeasy Kits for RNA Purification	Qiagen	74106		
Iq™ SYBR, 1708882	Bio-Rad	1708882		
CD19 Microbeads	Miltenyi Biotec	130-052-201		
Recombinant Mouse FLT3L (carrier-free)	Biolegend	550704		
Recombinant Mouse DLL1 Fc Chimera Protein, CF	R&D Systems	5026-DL-050		
DMA (N,N-Dimethylacetamide)	Sigma	CAS# 127-19-5 Cat. PHR2110		
PEG 400	Merck	8.17003.1000		
Cremophor	Sigma	C5135-500G		
Ficoll-Paque PLUS	Cytiva	17-1440-02		
Serologies				
MSD U-PLEX Custom Biomarker Group1	Meso Scale Diagnostics	K15069M-2		

**Table A2:** Antibodies.

Target	Clone	Fluorophore	Source	Cat Number	Dilution/ Concentration	RRID
Mouse Abs						
CD45	30-F11	AlexaFluor 700	Biolegend	103128	1:200	AB_493715
TCRß	H57 597	PE-Cyanine7	BD Biosciences	560729	1:200	AB_1937310
CD4	RM4_5	Pacific Blue	BD Biosciences	558107	1:100	AB_397030
CD8a	53-6.7	Qdot 605	BD Biosciences	563152	1:100	AB_2738030
CD44	IM7	Alexa Fluor 700	Biolegend	103026	1:200	AB_493713
CD25	PC61	PE	BD Biosciences	553866	1:200	AB_395101
FOXP3	FJK-16s	APC	eBioscience	17-5773-82	1:200	AB_469457
CD45R/B220	RA3 6B2	PB	BD Biosciences	558108	1:200	AB_397031
CD19	6D5	PE	Biolegend	115520	1:200	AB_313655
LY6G	1A8	PerCP/Cy5.5	Biolegend	127616	1:200	AB_1877271
CD11c	N418	BV711	Biolegend	117349	1:200	AB_2563905
CD11b	M1/70 (RUO)	BV605	BD Biosciences	563015	1:200	AB_2737951
MHC Class II (I-A/I-E)	M5/114.15.2	FITC	Thermo Scientific	11-5321-85	1:200	AB_465233
IL-10	JES5-16E3	FITC	Biolegend	505006	1:200	AB_315360
IgM, µ chain specific	Polyclonal		Jackson ImmunoResearch	115-006-075	1:200	AB_2338474
CD16/CD32 Fcr RIII	2.4G2		Holmdahl lab		1:50	
Human abs/PBMC ex vivo studies						
TCRβ	H57-597	AlexaFluor 488	Biolegend	109215	1:200	AB_493344
CD4	OKT4	BV605	Biolegend	317437	1:200	AB_11204077
CD25	M-A251	PE-Cy7	BD Biosciences	560920	1:200	AB_396847
FOXP3	PCH101	APC	eBioscience	17-4776-41	1:200	AB_1603281
IL10 anti-human	JES3-9D7	BV421	BD Biosciences	566276	1:200	AB_2738566
Fc Block	Fc1		BD Biosciences	564219	1:50	AB_2728082

**Table A3:** qPCR primers.

qPCR Targets	Primer Sequences (Source: Eurofins)		
	Forward	Reverse	Product Length (bp)
*B-actin*	GCAGGAGTACGATGAGTCCG	ACGCAGCTCAGTAACAGTCC	74
*Hmox1*	CAGAACCCAGTCTATGCCCC	GTGAGGCCCATACCAGAAGG	93
*Nqo1*	CTCACGGAAGTATGTGTCTCCT	CCCAGGGCTGAGGGTGTATT	105
*Srxn1*	CTCATACCTTCCAGTTTGGGT	AGGCTATTGCATGGTGTGT	149
*Txnrd1*	AGCGAGGAGACCATAGAGGG	CTCCAGGATGTCACCGATGG	186
*Xct*	AGCGAAGGCTGAAACACAC	CCCTTTGCTATCACCGACTGG	76
